# Visceral leishmaniasis & HIV co-infection in Bangladesh: Investigating prevalence and policy recommendations

**DOI:** 10.1016/j.jiph.2026.103233

**Published:** 2026-06

**Authors:** Faria Hossain, Ashik Sharfaraz, Prakash Ghosh, Golam Sarwar, Md. Rasel Uddin, Md. Utba Rashid, Debashis Ghosh, Rupen Nath, Shomik Maruf, Soumik Kha Sagar, Mohammad Sohel Shomik, Proggananda Nath, Abu Nayeem Mohammad Sohel, Md. Sakhawat Hossain, Sharful Islam Khan, Megha Raj Banjara, Christine M. Halleux, Abraham Aseffa, Dinesh Mondal

**Affiliations:** a Nutrition Research Division, International Centre for Diarrhoeal Disease Research (icddr,b), Dhaka, Bangladesh; b Programme for HIV and AIDS, Health Systems and Population Studies Division, International Centre for Diarrhoeal Disease Research (icddr,b), Dhaka, Bangladesh; c Mymensingh Medical College and Hospital (MMCH), Mymensingh, Bangladesh; d National Kala-azar Elimination Programme, CDC, DGHS, Bangladesh; e Institute for Animal Hygiene and Veterinary Public Health, Leipzig University, Leipzig, Germany; f Department of Empirical Health Economics, Technische Universität Berlin, Berlin, Germany; g Central Department of Microbiology, Tribhuvan University, Kirtipur, Kathmandu, Nepal; h UNICEF/UNDP/World Bank/World Health Organization Special Programme for Research and Training in Tropical Diseases (TDR), World Health Organization, Geneva, Switzerland; i Department of Health Policy and Management, College of Public Health, University of Georgia, Athens, GA, USA

**Keywords:** VL-HIV, RVL, PKDL, Co-infection, *Leishmania donovani*, Kala-azar surveillance, Case management

## Abstract

**Background:**

Human Immunodeficiency Virus (HIV) co-infection has emerged as a significant challenge in implementing the World Health Organization’s (WHO’s) strategic interventions to eliminate visceral leishmaniasis (VL) in co-endemic countries. VL-HIV co-infection results in rapid deterioration, higher mortality, and substantially increases the risk of developing VL, facilitating parasite transmission. Although Bangladesh has received official validation for the elimination of VL as a public health problem in 2023, determining the prevalence of VL-HIV co-infection became imperative to develop effective strategies to sustain the elimination status. Therefore, in this study, we aimed to investigate the prevalence of HIV infection in VL patients in Bangladesh.

**Methods:**

In this cross-sectional study, a total of 656 VL, post kala-azar dermal leishmaniasis (PKDL), and relapsed VL patients were enrolled. and tested for HIV following national HIV testing guidelines. Furthermore, the knowledge and attitudes of 624 participants were assessed through one-to-one interviews using structured questionnaires.

**Findings:**

None of the patients tested positive for VL-HIV co-infection. Only 9.46% of respondents demonstrated comprehensive HIV/AIDS knowledge and 40.06% had positive attitudes towards individuals living with HIV/AIDS. Age, educational status, and home visits by government healthcare workers showed significant positive associations with participants’ knowledge and attitudes in multivariate regression analysis.

**Conclusion:**

The study found no cases of HIV coinfection among the screened VL cohort in Bangladesh. However, the unavailability or response refusal of 26.1% of previous patients may have introduced significant selection bias. Therefore, considering the substantial health implications and increasing HIV incidence in the general population, we recommend continued routine HIV testing in all VL patients in Bangladesh, aligning with the WHO recommendations. Coordination between the respective national programs is warranted to develop effective strategies to detect emergence of such co-infected cases.

**Funding:**

This study was conducted with the financial support of the UNICEF/UNDP/World Bank/WHO Special Programme for Research and Training in Tropical Diseases (TDR).

Research in Context

Evidence before this study

Before conducting this study, we performed an extensive literature review using databases including PubMed, Scopus, and Web of Science. We searched using the terms “visceral leishmaniasis,” “VL-HIV co-infection,” “Bangladesh,” “Southeast Asia,” “Africa”, and “post-elimination surveillance.” No language or publication date restrictions were applied. Previous studies depicted that 70% of the global VL burden was concentrated in three South-East Asian countries-Bangladesh, India, and Nepal. Studies from the Indian subcontinent, particularly from India and Nepal, have documented the clinical and epidemiological impact of VL-HIV co-infection. These include atypical clinical presentations, poor response to antileishmanial treatment, increased relapse rates, and higher mortality. HIV greatly increases vulnerability to *Leishmania* infection and vice versa, making VL-HIV co-infection a serious obstacle to eradication initiatives, according to reports from East Africa. Furthermore, the HIV incidence in general population is increasing in Bangladesh, especially in the post-pandemic era. However, epidemiological data on VL-HIV co-infection in Bangladesh is not currently available.

Added value of this study

To the best of our knowledge, this is the first study to estimate the prevalence of HIV co-infection among VL patients in Bangladesh. This study is among the first to assess knowledge and attitudes in the general population of Bangladesh, with representation from both males and females in rural settings. Participants were screened for HIV in accordance with National HIV testing services guidelines, Bangladesh. No cases of VL-HIV co-infection were identified within the screened population, indicating the current low HIV burden among this population. Furthermore, we also assessed knowledge and attitudes towards HIV/AIDS among respondents using structured questionnaires. A very low percentage of respondents had comprehensive knowledge regarding HIV transmission and prevention, and below half of the participants expressed positive attitudes towards individuals living with HIV/AIDS.

Implications of all the available evidence

Although none of the participants reported VL-HIV co-infection in the study, we advocate for HIV testing of all VL patients in Bangladesh in adherence to the WHO guideline. Introducing such an intervention *earlier* will circumvent the reported severe health consequences of co-infection. Therefore, close coordination is warranted between the National Kala-azar Elimination Programme and the National AIDS/STD Programme for establishing guidelines on systematic surveillance, integrated case management, and preventative interventions.

## Introduction

Visceral leishmaniasis (VL) is a vector borne parasitic disease, endemic in large areas of the tropics, subtropics, and the Mediterranean Basin. In East Africa and the Indian sub-continent (ISC), it is caused by *Leishmania donovani* infection. In 2005, 70% of the global disease burden was reported from three countries in the World Health Organization’s (WHO) South Asian Region - Bangladesh, India, and Nepal. With a goal of decreasing the annual incidence to less than one per 10,000 population, these three countries signed a memorandum of Elimination of kala-azar by 2015. The memorandum was extended up to 2020 through the London declaration [1]. Bangladesh, through a collaborative effort between the government and public-private partnerships, achieved the elimination of VL in all formerly endemic districts by 2017. The country became the first to receive official validation for this accomplishment in 2023 globally [2].

With the reduced VL cases, the nation is now focused on optimizing strategies to sustain the elimination of kala-azar as a public health concern during the current post-validation era. One of the critical challenges affecting VL control efforts in the endemic countries is the rise of *Leishmania* as an opportunistic infection among people living with HIV (PLHIV) [3]. Since the first case documented in 1985 in Southern Europe, co-infection has spread across the globe, with at least 45 countries reporting VL-HIV cases in 2022 [4]. Co-morbidity of VL-HIV causes rapid deterioration and increased risk for death, as the pathogens complement each other’s replication by creating a shared immune-suppression-exhaustion niche. HIV infection can also promote activation and re-activation of latent *Leishmania* infection. Such co-infection elevates the rate of VL relapse and treatment failures. Reportedly, 25–61% of co-infected patients relapse after treatment [5], [6].

Due to their immunosuppressed state, PLHIV are also at a 100–2300 times greater risk of developing VL compared to non-HIV infected individuals [7]. Additionally, the parasite load in HIV co-infected VL patients is also significantly higher than that of non-HIV infected VL patients, making them potential reservoir of parasites. This greater infectiousness among co-infected patients is supported by xenodiagnostic studies. Notably, 45–93% of VL–HIV co-infected patients transmitted parasites to sandflies in the studies, representing a 2–3 fold increase compared with non-HIV-infected VL patients [7], [8]. The high parasite load observed in these immunosuppressed individuals, along with their greater infectiousness, poses a risk of ongoing transmission. This may lead to potential VL outbreaks, jeopardizing elimination efforts in co-endemic settings.

With an aim to sustain VL elimination as a public health problem, the WHO has identified three strategic priorities to achieve this goal: kala-azar and PKDL surveillance, complete case management, and integrated vector management [3]. The burden of VL-HIV co-infection is one of the critical determinants in implementing the relevant recommendations. This is especially crucial during the post-elimination phase, when the primary aim shifts toward limiting transmission and sustaining elimination. Additionally, the chronic, complex clinical condition of the co-infected patients necessitates specialized and coordinated approaches for diagnosis and treatment [4]. The epidemiology and prevalence of VL-HIV co-infection vary across different geographic regions and healthcare systems. Hence, implementation of these strategies is contingent on the country specific data on the co-infection status, epidemiology, and local health system capacity [3].

There are gaps in the current knowledge of VL-HIV co-infection burden in Bangladesh. Estimation of the VL-HIV co-infection burden is warranted to inform post-validation control strategies, strenghtnen referral approaches for co-infected patients, and to guide resource allocation. As such, to determine the status of VL-HIV co-infection in Bangladesh, in this study, a sero-surveillance for HIV infection was performed among VL, post kala-azar leishmaniasis (PKDL), and relapsed VL (RVL) patients in the major endemic regions in Bangladesh.

## Methods

### Study design, participant enrolment, and sample collection

A cross-sectional design was employed in the study. Participants in different Ethical Review Committee (ERC) approved studies (protocols PR-14093, PR-17041, PR-17116, PR-11001, PR-13045 and PR-14010) at International Centre For Diarrhoeal Disease Research, Bangladesh (icddr,b), or sought treatment for VL, PKDL or RVL at Surya Kanta Kala-azar Research Centre (SKKRC), Mymensingh between June 2016 and August 2023 were invited to participate in this study over phone using a pre-formed script. Participants who were willing to participate in the study visited SKKRC hospital for enrolment and sample collection.

VL, PKDL and RVL cases were defined according to the national guidelines [9]. An individual from a VL endemic area of either sex with no history of previous VL, suffering from fever for more than two weeks with splenomegaly and positive in rK39 RDT or, *Leishmania donovani* (LD) PCR was defined as a VL case. An individual from a VL endemic area of either sex, previously treated and cured for VL, suffering from fever for more than two weeks with splenomegaly, and positive in LD PCR was defined as a relapsed VL case. A treated VL case of either sex from VL endemic area presenting with skin lesion with preserved skin sensitivity and positive in rK39 RDT or, LD PCR was defined as a PKDL case [9], [10].

Consent or assent (with parental consent) through signature or thumbprint in the presence of a literate witness was obtained from study participants. All participants agreed to provide fresh sample for the study. Followed by pre-test HIV counselling, 1.5 mL venous blood was collected from the participants in plain blood collection tubes. The tubes were then centrifuged at 1258 × g for 10 min to separate the serum, and one aliquot of 200 uL serum was prepared in microcentrifuge tubes for subsequent laboratory analysis at SKKRC by the study team from icddr,b.

### Rapid detection tests for HIV

**Fig. 1:**
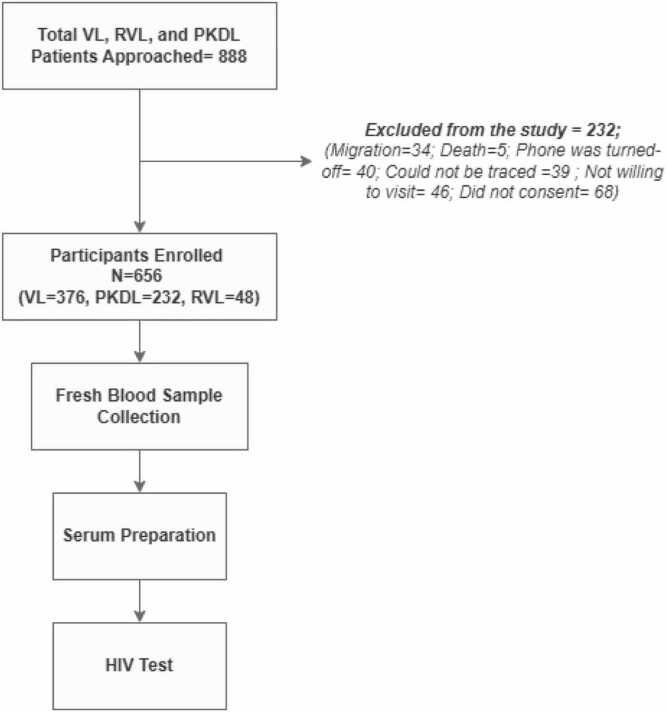
Study Activities Flow Diagram.

**Fig. 2:**
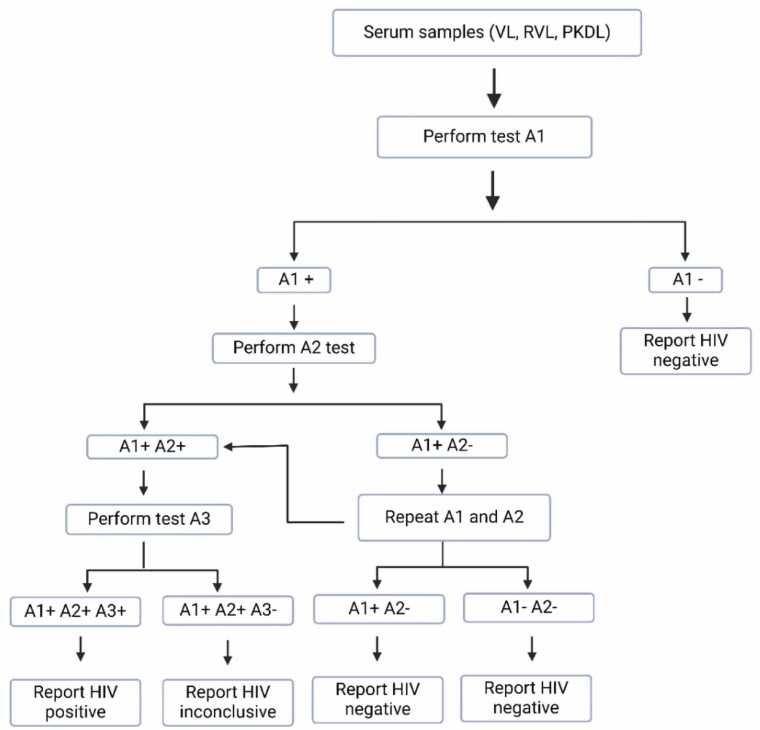
Laboratory test algorithm for HIV; A1 = Alere Determine HIV1/2 RDT, A2 = Uni-Gold TM Recombigen® HIV RDT, A3 = First Response HIV -1-2-0 RDT.

Laboratory testing for HIV was performed following the laboratory test algorithm (Fig. 1) outlined in the National HIV testing services guidelines, Bangladesh [11]. Firstly, serum/plasma samples were subjected to Alere Determine HIV-1/2 rapid detection test (RDT). Any samples that tested positive were then serially analyzed through Uni-Gold TM Recombigen® HIV and First Response HIV-1-2-0 RDTs according to the manufacturer’s instructions (Fig. 2).

### Assessment of risk factors of HIV/AIDS

The information of risk behavior was obtained from the pre-test counselling questionnaire according to HIV testing guidelines of AIDS/STD program, Bangladesh [11]. Comprehensive data on participants' prior HIV testing, non-sexual exposures such as nose or ear piercing and history of blood transfusion were obtained. Behavioral risks data like engagement in sex work and sharing needles or syringes were also collected. Additionally, exposure through partners' risky behaviors and partner/parent’s HIV infection were also assessed. Sexual practices were examined by recording the number and type of partners, condom use, and modes of sexual activities. Medical history was reviewed for previous diagnoses of sexually transmitted diseases and tuberculosis, providing an overview of factors influencing participants' health and HIV risk.

### Assessment of knowledge and attitude towards HIV/AIDS

A face-to-face interview of the consenting participants was performed for an assessment of their knowledge and attitude towards HIV/acquired immunodeficiency syndrome (AIDS). Data was collected using a pre-designed structured questionnaire in Bengali language. The comprehensive knowledge of HIV was defined according to the Joint United Nations Programme on HIV/AIDS (UNAIDS) guidelines, with respondents needing to answer five questions correctly to be considered knowledgeable: 1) “Can using condoms reduce the risk of HIV transmission?” (correct answer is ‘yes’), 2) “Can having sex with only one faithful, uninfected partner reduce the risk of HIV transmission?” (correct answer is ‘yes’), 3) “Can a person get HIV from mosquito bites?” (correct answer is ‘no’), 4) “Can a person get HIV by sharing a meal with someone who is infected?” (correct answer is ‘no’), and 5) “Can you tell by looking at a person if he or she has HIV?” (correct answer is ‘no’) [12]. In addition to assessment of comprehensive knowledge using the UNAIDS indicator to understand whether knowledge was sufficient, we further examined relative difference in knowledge across subgroups through a knowledge score. For comparison with other previously published findings from the region, the mean score (2.45) was used as the cut-off to further dichotomize respondents’ overall HIV/AIDS knowledge into high and low level of knowledge [13], [14]. Respondents with scores of 3–5 were coded as having "high level knowledge," while scores of 0–2 indicated having "low level knowledge". Respondents who answered *all* five questions correctly were considered to have “comprehensive knowledge”.

Similarly, attitudes toward people living with HIV/AIDS were assessed using a set of eight questions. The questions were guided by the “Health Stigma and Discrimination (HSD) Framework” for general population [15]. The eight questions used in the study were selected according to the HSD framework to comprehensively assess attitudes related to HIV/AIDS. These questions addressed four core domains: Social Judgment (willingness to care for a relative with HIV/AIDS and whether the participant has ever refused care to someone with HIV/AIDS), Experienced Stigma (maintaining a friendship with an HIV-positive individual and willingness to buy from an HIV-positive shopkeeper or food seller), Discrimination (support for allowing HIV-positive students and teachers to continue their education or teaching), and Fear of Infection (comfort with sharing eating or drinking utensils with a person living with HIV) [15], [16]. Incorrect answers and "don't know" responses were coded as 0, while correct answers were coded as 1. Each respondent's overall attitude score ranged from 0 to 8. The mean score (3.42) was used as a cut-off to dichotomize the attitude scores into positive and negative attitudes. Respondents with scores of 4–8 were classified as having a “positive attitude”, while those with scores of 0–3 were classified as having a “negative attitude” towards HIV/AIDS [14].

### Statistical analysis

Parametric and nonparametric tests were performed based on the distribution of data. The chi-square test, or fisher exact test determined relationships within demographic features among patient groups. Bivariate binary logistic regression analyses were conducted to examine associations between independent variables and participants’ overall knowledge of HIV/AIDS and attitudes toward PLHIV. Variables were subsequently entered into multivariable logistic regression models to identify factors independently associated with each outcome [14]. All statistical analyses were performed using STATA (Stata Statistical Software: Release 16, College Station, Texas, USA: StataCorp LLC) and GraphPad Prism (version 9.1). Python (Version 3.12.0) software was used to develop case density maps for Bangladesh. A P-value < 0.05 was considered statistically significant.

## Results

**Fig. 3:**
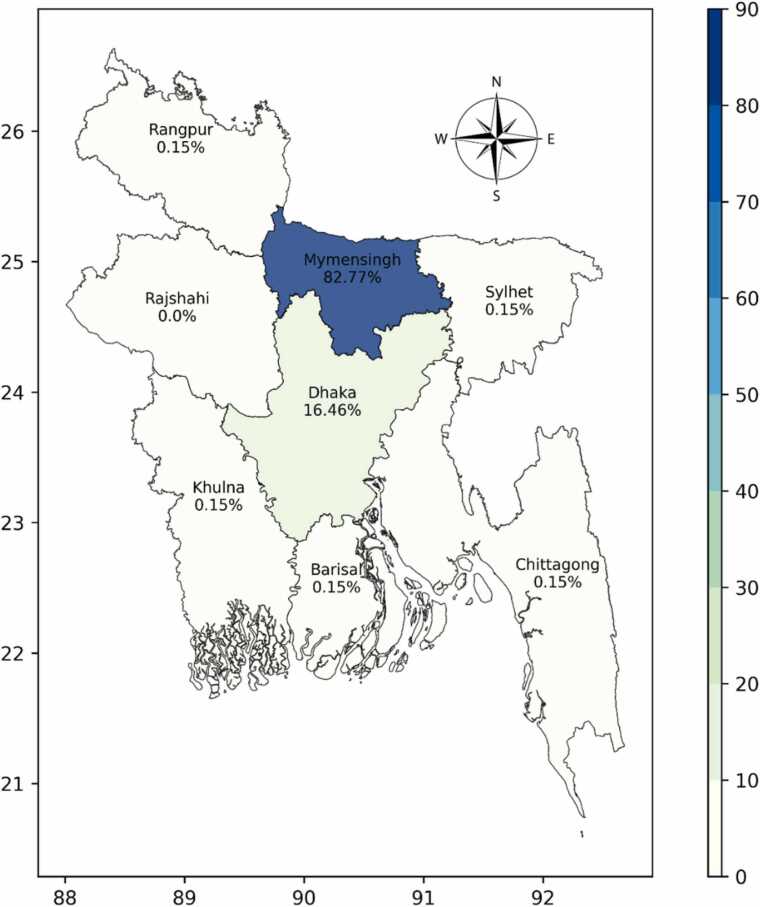
Geographic distribution of the enrolled participants in the study.

A total of 656 participants, aged 9–75 years, were recruited between 25 December 2022 and 27 August, 2023. This represents 74% of the former VL patients approached. The majority, 82.77% (543/656) of the participants in the study were from the Mymensingh district, followed by Dhaka district, at 16.46% (108/656) (Fig. 3). Our study included participants representative of 41% of the total VL/ RVL cases and 35% of the total PKDL cases reported nationally between 2016 and 2023.^1^ Notably, 26.1% (238/888) of previous patients were not enrolled due to unavailability, death or refusal to provide consent.

### Socio-demographic characteristics

The socio-demographic characteristics of the study participants are presented in Table 1. The mean age of the relapsed VL patients was significantly higher (p = 0.011) compared to the VL and PKDL patients, though there was no significant difference in the first VL episode across the groups. Majority of the participants across all groups were male. Close to half (46.49%) of the participants had completed secondary or higher levels of education. The primary occupations of the participants were household management (26.52%) and agriculture (18.45%). Most participants, 95.58%, resided in rural areas. Additionally, 59.30% of the participants had more than four family members, and 97.87% of the participants were accustomed to seeking healthcare at the nearest medical facility.

**Table 1:** Socio-demographic characteristics of the study participants.

Indicators	VL% (n)	PKDL% (n)	Relapsed VL% (n)	Total% (n)
No. of participants (N)	376	232	48	656
Age in years, Mean±SD	32.11 ± 15.51	32.95 ± 15.54	39.31 ± 17.25	32.93 ± 15.74
Age at 1st VL episode in years, Mean±SD)	22.86 ± 16.23	20.63 ± 17.73	25.56 ± 16.34	22.27 ± 15.76
Gender of the participants				
Male	60.90 (229)	62.50 (145)	75.00 (36)	62.50 (410)
Female	39.10 (147)	37.50 (87)	25.00 (12)	37.50 (246)
Marital status				
Married	68.62 (258)	74.14 (172)	85.42 (41)	71.80 (471)
Never married	27.93 (105)	24.57 (57)	12.50 (6)	25.61 (168)
Widow/Widower	1.60 (6)	1.29 (3)	2.08 (1)	1.52 (10)
Divorced/Separated	1.86 (7)	0.00 (0)	0.00 (0)	1.06 (7)
Age at marriage in years, Mean±SD	18.96 ± 4.52	19.51 ± 4.76	19.60 ± 4.40	19.21 ± 4.60
Educational status				
Illiterate	26.33 (99)	28.02 (65)	27.08 (13)	26.98 (177)
Primary	25.80 (97)	25.43 (59)	37.50 (18)	26.52 (174)
Secondary	36.44 (137)	32.76 (76)	29.17 (14)	34.60 (227)
Higher and above	11.44 (43)	13.79 (32)	6.25 (3)	11.89 (78)
Occupation				
Housewife	26.60 (100)	26.72 (62)	25.00 (12)	26.52 (174)
Agriculture	16.49 (62)	17.67 (41)	37.50 (18)	18.45 (121)
Job in office	17.02 (64)	17.67 (41)	10.42 (5)	16.77 (110)
Student	17.29 (65)	14.66 (34)	4.17 (2)	15.40 (101)
Business	11.17 (42)	9.05 (21)	2.08 (1)	9.76 (64)
Labor	5.05 (5)	5.17 (12)	6.25 (3)	5.18 (34)
Others	6.38 (24)	9.05 (21)	14.58 (7)	7.93 (52)
Number of people in the family				
> 4	59.31 (223)	62.07 (144)	45.83 (22)	59.30 (389)
≤ 4	40.69 (153)	37.93 (88)	54.17 (26)	40.70 (267)
Monthly family income (in BDT) Median (IQR)	15,500 (12,000–22,000)	15,000 (12,000–20,000)	16,000 (12,000–20,000)	15,000 (12,000–20,000)
Living area				
Rural	95.21 (358)	96.55 (224)	93.75 (45)	95.58 (627)
Urban	3.19 (12)	2.16 (5)	4.17 (2)	2.90 (19)
Semi-urban	1.60 (6)	1.29 (3)	2.08 (1)	1.52 (10)
Regularly read the newspaper	7.71 (29)	5.60 (13)	4.17 (2)	6.71 (44)
Regularly watch television	40.16 (151)	39.22 (91)	29.17 (14)	39.02 (256)
Visits nearest health care center for illness	97.34 (366)	98.71 (229)	97.92 (47)	97.87 (642)
Type of health care center				
Upazila health complex	51.91 (190)	64.63 (148)	59.57 (28)	57.01 (366)
Community clinic	20.77 (76)	15.72 (36)	12.77 (6)	18.38 (118)
District hospital	13.93 (51)	11.35 (26)	10.64 (5)	12.77 (82)
Private hospital	13.39 (49)	8.30 (19)	17.02 (8)	11.84 (76)
Treatment History (n = 642)				
Single dose liposomal amphotericin B	71.73 (269)	1.32 (3)	12.77 (6)	42.77 (278)
Multiple doses liposomal amphotericin B	23.73 (89)	26.75 (61)	85.11 (40)	29.23 (190)
Miltefosine Monotherapy	3.73 (14)	69.74 (159)	2.13 (1)	26.77 (174)

## Prevalence of HIV in VL, PKDL and RVL patients

All participants were tested negative by the HIV testing algorithm.

### HIV risk behaviors

The risk behavior, knowledge and attitude towards HIV/AIDS were assessed in 624 participants aged 15–75. Risk behavior for HIV was observed to be minimal for most of the indicators except for use of condom, as 80.62% of the participants having at least one sexual partner were accustomed to unprotected sex.Only 4.54% of participants reported using condom regularly. 33.81% of the participants reported to have ear piercing whereas 10.10% of participants had received blood transfusion (Table 2).

**Table 2:** Risk behaviors of the study participants.

Indicators	Total %(n)
No. of participants (N)	624
Ever tested for HIV	0.00 (0)
Level of the risk of the study participant	
Nose/ear piercing or tattooing on any part of the body	33.81 (211)
Blood transfusion	10.10 (63)
Sex workers	2.24 (14)
Partner is accustomed to risky behavior	0.48 (3)
Share needles/syringes	0.00 (0)
Partner/parent is infected with HIV	0.00 (0)
No. of the sexual partner	
One	74.52 (465)
None	22.28 (139)
More than One	3.21 (20)
Type of the partners (n = 485)	
Regular	95.67 (464)
Irregular	4.33 (21)
Use of condom (n = 485)	
None	80.62 (391)
Irregular	14.85 (72)
Regular	4.54 (22)
Mode of sexual activities (n = 485)	
Vagina	98.97 (480)
Anal	0.62 (3)
Oral	0.41 (2)
Diagnosed with STD	3.37 (21)
Diagnosed with TB	3.53 (22)

### Knowledge of HIV/AIDS and attitude towards PLHIV

**Fig. 4:**
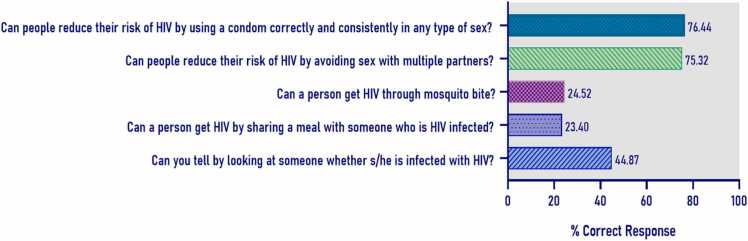
Respondents knowledge of HIV/AIDS.

In our study, 9.46% (59/624) respondents demonstrated a comprehensive knowledge about HIV. Of the respondents, 76.44% (477/624) and 75.32% (470/624) participants correctly answered questions on the role of using protection during sex act and sexual behaviors, respectively, in reducing HIV risk (Fig. 4). In contrast, the lowest proportions of correct response were observed for questions assessing knowledge about the transmission of HIV through shared meals, at 23.40% (146/624) and mosquito bites, at 24.52% (153/624). Furthermore, less than half of the respondents, at 44.87% (280/624), correctly responded that HIV-infected individuals could not be visually identified.

Following dichotomization however, 52.08% (325/624) of the respondents demonstrated a high knowledge level. The level of knowledge varied significantly with respondents’ age (p = <0.001), marital status (p = 0.035), education (p = <0.001), occupation (p = <0.001), newspaper reading (p = 0.002), television viewing (p = <0.001) and household visits by public healthcare providers (p = 0.001) (Table 3).

**Table 3:** Factors influencing knowledge of HIV/AIDS and attitude towards PLHIV within respondents.

Indicators	Knowledge		p-value	Attitude		p-value
	**Low% (n)**	**High% (n)**		**Negative% (n)**	**Positive% (n)**	
Age (in years), Mean±SD	35.02 ± 17.18	33.03 ± 13.54	0.107	36.18 ± 16.35	30.70 ± 12.69	**< 0.001**
Age (in years)						
15–19	61.72 (79)	38.28 (49)	**< 0.001**	62.50 (80)	37.50 (48)	**< 0.001**
20–29	37.89 (72)	62.11 (118)		45.79 (87)	54.21 (103)	
30–39	35.00 (28)	65.00 (52)		57.50 (46)	42.50 (34)	
40–49	35.96 (32)	64.04 (57)		59.55 (53)	40.45 (36)	
50–59	62.96 (51)	37.04 (30)		76.54 (62)	23.46 (19)	
≥ 60	66.07 (37)	33.93 (19)		82.14 (46)	17.86 (10)	
Gender of the participants						
Male	45.45 (180)	54.55 (216)	0.105	60.86 (241)	39.14 (155)	0.535
Female	52.19 (119)	47.81 (109)		58.33 (133)	41.67 (95)	
Marital status						
Married	45.70 (233)	54.30 (265)	**0.035**	59.63 (291)	40.37 (197)	0.769
Unmarried	55.88 (76)	44.12 (60)		61.03 (83)	38.97 (53)	
Educational status						
Higher and above	20.78 (16)	79.22 (61)	**< 0.001**	28.57 (22)	71.43 (55)	**< 0.001**
Secondary	42.52 (91)	57.48 (123)		51.40 (110)	48.60 (104)	
Primary	48.72 (76)	51.28 (80)		66.03 (103)	33.97 (53)	
Illiterate	65.54 (116)	34.46 (61)		78.53 (139)	21.47 (38)	
Occupation						
Desk Job	31.82 (35)	68.78 (75)	**< 0.001**	54.55 (49)	55.45 (61)	**0.008**
Business	34.38 (22)	65.63 (42)		36.55 (56)	43.75 (28)	
Labor	44.12 (15)	55.88 (19)		64.71 (22)	35.29 (12)	
Agriculture	52.89 (64)	47.11 (57)		66.12 (80)	33.88 (41)	
Housewife	53.76 (93)	46.24 (80)		60.12 (104)	39.88 (69)	
Student	56.34 (40)	43.66 (31)		64.79 (46)	35.21 (25)	
Number of people in the family						
≤ 4	48.85 (127)	51.15 (133)	0.694	54.62 (142)	45.38 (118)	**0.022**
> 4	47.25 (172)	52.75 (192)		63.74 (232)	36.26 (132)	
Residency						
Urban	50.00 (14)	50.00 (14)	0.821	60.71 (17)	39.29 (11)	0.931
Rural	47.82 (285)	52.18 (311)		59.90 (357)	40.10 (239)	
Regularly read the newspaper	25.00 (11)	75.00 (33)	**0.002**	43.18 (19)	56.82 (25)	**0.019**
Regularly watch television	37.60 (91)	62.40 (151)	**< 0.001**	52.89 (128)	47.11 (114)	**0.004**
Visits nearest health center for illness	47.70 (291)	52.30 (319)	0.485	59.84 (365)	40.16 (245)	0.791
Govt. health worker visits home	41.89 (142)	58.11 (197)	**0.001**	55.75 (189)	44.25 (150)	**0.020**
Private health worker visits home	45.65 (168)	54.35 (200)	0.175	59.24 (218)	40.76 (150)	0.670

**Fig. 5:**
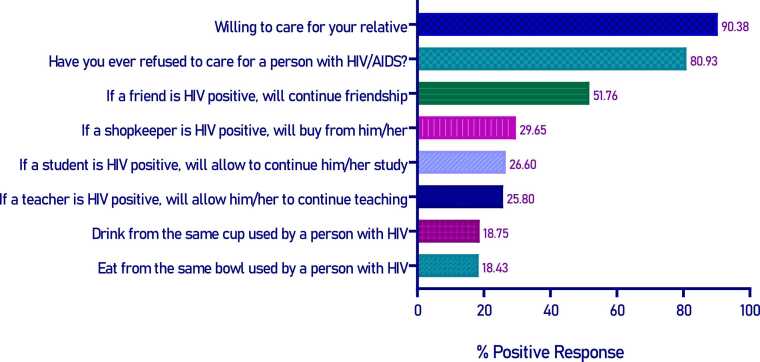
Respondents attitude towards HIV/AIDS.

Among the respondents, only 40.06% (250/624) showed a positive attitude towards PLHIV following dichotomization. A higher proportion of positive response was recorded for the questions regarding willingness to provide care for their relatives affected by HIV/AIDS and other people, at 90.38% (564/624) and 80.93% (505/624) respectively (Fig. 5). About half of the participants, 51.76% (323/624), indicated they would maintain friendships with PLHIV. However, a lower percentage of participants expressed willingness to share utensils, such as drinking from the same cup at 18.75% (117/624) and eating from the same bowl at 18.43% (115/624). Approximately three-quarters of the participants were reluctant to engage in day-to-day interactions with PLHIV such as grocery shopping, and education. A significantly higher percentage of positive attitude was observed in young adults (p = <0.001), higher education (p = <0.001), desk job & business (p = 0.008), newspaper readers ((p = <0.019), television viewers (p = <0.004), household size (p = 0.022), and household visits by public healthcare providers (p = <0.020) (Table 3).

### Factors associated with knowledge level and attitude towards PLHIV

In bivariate analysis, age group 20–49 years was significantly associated with knowledge of HIV (p < 0.001) (Table 4). After adjusting in multivariate logistic regression, respondents aged 20–29 years (AOR = 2.10; 95% CI: 1.10–4.01; p = 0.024), 30–39 years (AOR = 3.11; 95% CI: 1.37–7.05; p = 0.007), and 40–49 years (AOR = 3.87; 95% CI: 1.68–8.92; p = 0.002) were significantly more likely to have higher levels of HIV knowledge. Educational status was also significantly associated with knowledge in both bivariate and multivariate analyses. After adjustment, respondents with primary education (AOR = 2.40; 95% CI: 1.41–4.08; p = 0.001), secondary education (AOR = 4.03; 95% CI: 2.29–7.07; p < 0.001), and higher education or above (AOR = 10.71; 95% CI: 4.74–24.16; p < 0.001) were significantly more likely to have higher knowledge levels. Participants who reported government health worker home visits were also significantly more likely of having knowledge (AOR = 2.66; 95% CI: 1.53–4.62; p = 0.001). Although exposure to television and newspapers was significantly associated with knowledge level in bivariate analysis (p = 0.002 and p < 0.001, respectively), these associations were no longer significant after adjustment in the multivariate regression model.

**Table 4:** Bivariate and multivariate logistic regression analysis of knowledge level among respondents.

Indicators	Knowledge level					
	Bivariate			Multivariate		
	COR	CI (95%)	p-value	AOR	CI (95%)	p-value
Age (in years)						
15–19	1			1		
20–29	2.64	1.67–4.19	**< 0.001**	2.10	1.10–4.01	**0.024**
30–39	2.99	1.67–5.36	**< 0.001**	3.11	1.37–7.05	**0.007**
40–49	2.87	1.64–5.03	**< 0.001**	3.87	1.68–8.92	**0.002**
50–59	0.95	0.53–1.69	0.857	1.46	0.61–3.51	0.400
≥ 60	0.83	0.43–1.60	0.574	1.15	0.44–3.00	0.772
Gender of the participants						
Male	1			1		
Female	0.76	0.55–1.06	0.105	0.94	0.46–1.92	0.874
Marital status						
Unmarried	1.			1		
Married	1.51	1.03–2.21	**0.036**	1.90	0.93–3.90	0.078
Educational status						
Illiterate	1			1		
Primary	2.00	1.29–3.11	**0.002**	2.40	1.41–4.08	**0.001**
Secondary	2.57	1.70–3.88	**< 0.001**	4.03	2.29–7.07	**< 0.001**
Higher and above	7.25	3.85–13.64	**< 0.001**	10.71	4.74–24.16	**< 0.001**
Occupation						
Labor	1			1		
Agriculture	0.70	0.33–1.51	0.367	0.81	0.33–1.96	0.636
Business	1.51	0.64–3.53	0.345	0.97	0.37–2.53	0.947
Desk Job	1.69	0.77–3.72	0.190	1.06	0.43–2.62	0.896
Housewife	0.68	0.32–1.42	0.305	0.50	0.18–1.43	0.197
Student	0.61	0.27–1.39	0.242	0.74	0.24–2.35	0.613
Number of people in the family						
≤ 4	1			1		
> 4	1.07	0.78–1.47	0.694	1.30	0.90–1.88	0.158
Residency						
Rural	1			1		
Urban	0.92	0.43–1.96	0.821	0.76	0.31–1.89	0.556
Regularly read the newspaper						
No	1			1		
Yes	2.96	1.47–5.97	**0.002**	1.23	0.56–2.73	0.607
Regularly watch television						
No	1			1		
Yes	1.98	1.43–2.76	**< 0.001**	1.34	0.92–1.97	0.131
Visits nearest health center for illness						
No	1			1		
Yes	1.46	0.50–4.26	0.487	1.21	0.34–4.28	0.770
Govt. health worker visits home						
No	1			1		
Yes	1.70	1.24–2.34	**0.001**	2.66	1.53–4.62	**0.001**
Private health worker visits home						
No	1			1		
Yes	1.25	0.91–1.72	0.175	0.59	0.34–1.03	0.062

Similarly, age group was significantly associated with attitude toward HIV in bivariate analysis (p = 0.003) (Table 5). However, after adjustment, only respondents aged ≥ 60 years were significantly associated and were less likely to have a positive attitude (AOR = 0.27; 95% CI: 0.09–0.76; p = 0.013). Educational status was also significantly associated with attitude in bivariate analysis (p < 0.001). After adjusting, respondents with secondary education (AOR = 3.33; 95% CI: 1.90–5.84; p < 0.001) and higher education or above (AOR = 10.82; 95% CI: 4.99–23.43; p < 0.001) were significantly more likely to have a positive attitude. Family size was significantly associated with attitude in both bivariate (p = 0.022) and multivariate analyses, where respondents from families with more than four members were less likely to have a positive attitude (AOR = 0.69; 95% CI: 0.48–0.99; p = 0.046). Participants who reported government health worker home visits were more likely to have a positive attitude (AOR = 2.68; 95% CI: 1.51–4.76; p = 0.001), whereas those reporting private health worker home visits were less likely to have a positive attitude (AOR = 0.47; 95% CI: 0.27–0.84; p = 0.010). Although reading newspapers (p = 0.021) and watching television (p = 0.004) were significantly associated with attitude in bivariate analysis, these associations were not significant after adjustment in the multivariate regression model.

**Table 5:** Bivariate and multivariate logistic regression analysis of attitude levels among respondents.

Indicators	Attitude					
	Bivariate			Multivariate		
	COR	CI (95%)	p-value	AOR	CI (95%)	p-value
Age (in years)						
15–19	1			1		
20–29	1.97	1.25–3.12	**0.004**	1.20	0.64–2.25	0.576
30–39	1.23	0.70–2.18	0.473	0.83	0.38–1.84	0.645
40–49	1.13	0.65–1.97	0.661	0.85	0.38–1.92	0.704
50–59	0.51	0.27–0.96	**0.035**	0.42	0.17–1.03	0.058
≥ 60	0.36	0.17–0.78	**0.010**	0.27	0.09–0.76	**0.013**
Gender of the participants						
Male	1			1		
Female	1.11	0.80–1.55	0.535	1.26	0.63–2.53	0.520
Marital status						
Unmarried	1			1		
Married	1.06	0.72–1.56	0.769	1.41	0.70–2.83	0.338
Educational status						
Illiterate	1			1		
Primary	1.88	1.15–3.07	**0.011**	1.58	0.90–2.77	**0.111**
Secondary	3.46	2.21–5.41	**< 0.001**	3.33	1.90–5.84	**< 0.001**
Higher and above	9.14	4.96–16.85	**< 0.001**	10.82	4.99–23.43	**< 0.001**
Occupation						
Labor	1			1		
Agriculture	0.94	0.42–2.09	0.878	1.77	0.70–4.44	0.227
Business	1.43	0.60–3.37	0.418	1.19	0.45–3.14	0.725
Desk Job	2.28	1.03–5.07	**0.043**	1.12	0.46–2.74	0.807
Housewife	1.22	0.57–2.62	0.616	0.80	0.27–2.31	0.673
Student	1.00	0.42–2.34	0.993	0.44	0.14–1.42	0.171
Number of people in the family						
≤ 4	1			1		
> 4	0.68	0.49–0.95	**0.022**	0.69	0.48–0.99	**0.046**
Residency						
Rural	1			1		
Urban	0.97	0.44–2.10	0.931	0.94	0.38–2.31	0.888
Regularly read the newspaper						
No	1			1		
Yes	2.08	1.12–3.86	**0.021**	0.97	0.47–1.98	0.926
Regularly watch television						
No	1			1		
Yes	1.61	1.16–2.24	**0.004**	1.18	0.80–1.73	0.408
Visits nearest health center for illness						
No	1			1		
Yes	1.21	0.40–3.65	0.737	1.06	0.30–3.73	0.923
Govt. health worker visits home						
No	1			1		
Yes	1.47	1.06–2.03	**0.020**	2.68	1.51–4.76	**0.001**
Private health worker visits home						
No	1			1		
Yes	1.07	0.77–1.49	0.670	0.47	0.27–0.84	**0.010**

## Discussion

Identification of the VL-HIV co-infected patients is crucial to limit VL transmission by ensuring timely treatment, secondary prophylaxis, and protective interventions. In synergy, early diagnosis also ensures rapid initiation of antiretroviral therapy (ART) to achieve an undetectable viral load in the co-infected patients. Therefore, VL-HIV management requires a holistic, integrated approach for screening and surveillance in the co-endemic regions. The WHO recommends screening all HIV positive patients in VL endemic areas for VL, and testing all VL patients for HIV at primary care [3]. Due to high co-infection rates in India (7–20%) and Nepal (18%), the countries have implemented WHO-recommended guidelines, offering HIV testing to all VL-diagnosed individuals [5].

However, till date, a substantial knowledge gap persists regarding VL-HIV co-infection status in Bangladesh. Neither the National Kala-azar Elimination Programme (NKEP) nor National AIDS/STD Programme (NASP) has guidelines to address VL-HIV co-infection and manage co-infected patients in Bangladesh. Therefore, in this very first study, we aimed to estimate the HIV co-infection rate within the VL community in Bangladesh. A vast majority of the participants in our study were from Mymensingh district, a high VL burden district, accounting for over 60% of annual VL cases [17]. The district is also one of the 23 priority districts for HIV prevention [18]. Our study did not reveal any evidence of HIV co-infection in the VL, PKDL, or RVL patients within this screened patient cohort in Bangladesh.

For designing comprehensive and contextual prevention frameworks, it is crucial to understand the knowledge and awareness level of the at-risk population. The *comprehensive* knowledge was substantially lower similar to a previous study demonstrating a very low comprehensive knowledge of HIV and AIDS (3%) in the general population [19]. While only 9.46% met the stringent definition of comprehensive HIV knowledge, mean-based analyses revealed relative differences in knowledge levels across subgroups. The knowledge level about HIV in this community in our study was demonstrated to be lower compared to previous research within similar groups of rural male (58.6%) [19] and female (63.9%) populations [20]. The findings indicate that although most respondents lack complete knowledge, partial knowledge is unevenly distributed. Furthermore, more than 75% of the participants in our study displayed misconceptions about HIV transmission, believing it can spread through mosquito bites or shared meals. In comparison, previous research found lower rates of such misconceptions on transmission through mosquito bites or shared meals, 62.9% and 57.3% among rural women, and 46.8% and 45.9% among rural men [19], [20].

While assessing attitude towards HIV/AIDS affected individuals, higher proportion of participants demonstrated a negative attitude towards PLHIV. The attitude level is even lower compared to previous studies on reproductive-aged women in Bangladesh (45.8%), and urban slum dwellers (81.06%) [13]. However, respondents demonstrated higher positive attitude in terms of care for patients and family members aligned with previous studies [13]. A lower level of positive attitudes was observed regarding sharing utensils, likely due to misconceptions about casual contact transmission, as indicated in the knowledge assessment. The proportion of participants not using barrier protection during sex was higher than previously observed in the general population [13]. This study identified educational status as the strongest and most consistent predictor of both HIV knowledge levels and positive attitudes towards PLHIV, with higher education strongly associated with better outcomes, consistent with prior studies in Bangladesh [19]. Similar to previous studies, age also played an important role. Adults aged 20–49 years demonstrated higher knowledge levels [19], whereas individuals aged ≥ 60 years were *less* likely to hold positive attitudes. This observation may reflect generational differences in awareness and stigma. Respondents from larger households (>4 members) demonstrated a trend towards less favorable attitudes. This observation may possibly reflect more limited access to education or social contacts that reinforce stigmatizing beliefs.

Government health workers’ home visits were associated with both positive knowledge and attitudes, underscoring the effectiveness of structured public health outreach. Although media exposure showed associations in bivariate analyses, these effects were attenuated after adjustment, suggesting that passive media consumption alone is insufficient and largely confounded by education and age. In the knowledge assessment, only 9.78% of respondents correctly identified all non-transmission routes (mosquito bites, sharing meals, and casual contact). This indicates fragmented knowledge informed largely by fear-based misconceptions, which are also reflected in attitude levels. In genarl, misbeliefs regarding transmission through mosquito bites, sharing food, or external appearance remain widespread in Bangladesh and Southeast Asia [21], [22], [23]. Such misbeliefs are strongly associated with stigma toward PLHIV. Qualitative research is needed to inform policy by exploring fragmented knowledge and socio-cultural drivers of HIV-related stigma in the general population.

Based on these findings, several programmatic recommendations are warranted: integrate comprehensive HIV/AIDS education into school curricula, implement targeted programs for older adults and expand community education by trained government healthcare workers [19], [24]. One of NASP’s key strategies is to optimize behavior change communication to enhance case detection and reduce risk behaviors in the general population [18]. Effective communication must avoid stigmatizing or sensationalized portrayals and should not depict PLHIV solely as patients [25], [26]. Such targeted communication tools should be developed to ensure access to accurate information, correct misconceptions, and reduce fear and stigma. General messaging is also needed. Such sensitization efforts should include mass media messages, as well as group specific messages tailored for peer to peer sharing on social platforms.

Our study has limitations. First, the HIV status of 26.1% of the patients from previous studies who could not be traced, did not consent to participation or were dead remains unknown. Those who are HIV-positive may have been more likely to decline participation. Likewise, VL patients with HIV may have died or were untraceable. This gap may have potentially biased prevalence estimates downward. Such underestimation is a recognized challenge in population-based HIV surveillance. Individuals aware of their positive status may avoid testing due to fear of disclosure, stigma, or the belief that their status is already known [27], [28]. Additionally, the reported sexual behavior data of unmarried adolescents may have been subject to bias due to the presence of their parents during counseling sessions. Finally, differences in the analysis results regarding attitude level towards PLHIV, compared to previous studies, can be attributed to the lack of a standardized, universal questionnaire for assessing attitude towards PLHIV.

## Conclusion & Recommendation

In this study none of the participants demonstrated HIV co-infection within the largest VL cohort in Bangladesh. However, approximately 26% of registered patients were not tested for HIV. This untested subgroup may include individuals with undiagnosed HIV infection, including those who may have died before testing could be performed. As a result, HIV co-infection may be underestimated in our analysis creating potential selection bias. Given the high regional prevalence of VL–HIV co-infection, documented treatment failures, and the severe consequences of VL in immunocompromised patients, we advocate for routine HIV testing for all VL patients in Bangladesh. This approach aligns with WHO recommendations and global VL control strategies [3]. The post-pandemic rise of HIV incidence in the general population and evolving transmission dynamics in Bangladesh further underscores the need for universal HIV testing among VL patients [29]. Additionally, as the HIV knowledge in the VL community resembles low comprehensive knowledge within the general population, the NKEP platform can be leveraged to deliver education addressing misconceptions about HIV and promoting safer sexual practices in the general population, with a particular emphasis on adolescents. Therefore, robust coordination between the NKEP and NASP is warranted to support systematic national surveillance, integrated case-management, cross-reporting, and delivery of targeted interventions.

## Author Contributions

FH- Conceptualization, formal analysis, funding acquisition, investigation, methodology, project administration, supervision, validation, visualization, writing – original draft.

AS- Data curation, formal analysis, writing – original draft, writing – review & editing

PG- Conceptualization, funding acquisition, project administration, validation, writing – review & editing

GS- Resources, validation, writing – review & editing

MRU- Data curation, formal analysis, software, visualization, writing – review & editing

MUR- Project administration, methodology, supervision, writing – review & editing

DG- Project administration, methodology, supervision, writing – review & editing

RN- Data curation, investigation, writing – review & editing

SM- Project administration, supervision, writing – review & editing

SKS- Project administration, supervision, writing – review & editing

MSS- Project administration, supervision, writing – review & editing

PN- Resources, validation, writing – review & editing

ANMS- Resources, validation, writing – review & editing

MSH- Resources, validation, writing – review & editing

SIK- Resources, validation, writing – review & editing

MRB- Conceptualization, funding acquisition, resources, validation, writing – review & editing

CMH- Conceptualization, funding acquisition, resources, validation, writing – review & editing

AA- Conceptualization, funding acquisition, resources, validation, writing – review & editing

DM- Conceptualization, funding acquisition, resources, supervision, validation, visualization, writing – review & editing.

## Ethics statement

This study received ethical approval from the Institutional Review Board and Ethical Review Committee of the International Centre for Diarrhoeal Disease Research, Bangladesh (icddr,b) with protocol no. PR-20041. The study also received ethical approval from the Research Ethics Review Committee of the World Health Organization (WHO) with Protocol ID: ERC.0003608. Adhering to the Declaration of Helsinki, written informed consents were obtained from all participants or legal guardians of minors for the use of collected samples in research [30].

## Declaration of Generative AI and AI-assisted technologies in the writing process

During the preparation of this work the author(s) used Jenni.AI in order to improve readability. After using this tool/service, the author reviewed and edited the content as needed and take full responsibility for the content of the publication.

## Funding

This study was conducted with the financial support of the UNICEF/UNDP/World Bank/WHO Special Programme for Research and Training in Tropical Diseases (TDR).

## Declaration of Competing Interest

The authors declare no competing interests.

## Data Availability

The corresponding author can provide the raw data that explains the findings and study protocol upon reasonable request.

## Footnotes

1 Global Health Observatory data repository, WHO and DHIS2, NKEP database; 31 March, 2025.
